# Bilateral Multifocal Central Serous-Like Chorioretinopathy due to MEK Inhibition for Metastatic Cutaneous Melanoma

**DOI:** 10.1155/2013/673796

**Published:** 2013-03-11

**Authors:** Scott D. Schoenberger, Stephen J. Kim

**Affiliations:** Department of Ophthalmology, Vanderbilt Eye Institute, 2311 Pierce Avenue, Nashville, TN 37232, USA

## Abstract

Newer chemotherapeutic agents target extracellular signaling, including the mitogen-activated protein kinase kinase (MEK) pathway. We present a case of a 54-year-old female who developed bilateral multifocal central serous-like chorioretinopathy shortly after starting MEK inhibition for metastatic cutaneous melanoma. There was a complete resolution of findings after drug stoppage. After resuming a lower dose of the MEK inhibitor, the findings recurred again but resolved after drug stoppage. Other etiologies were unlikely given the clinical course. The presumed mechanism involves toxicity to the retinal pigment epithelium, with breakdown of the blood-retinal barrier. Recognition of this side effect is important with this new class of chemotherapy.

## 1. Introduction

Recently developed chemotherapeutic agents target the mitogen-activated protein kinase kinase (MEK) pathway [1]. Central serous-like chorioretinopathy has been reported with different MEK inhibitors [1–3], but detailed information about the retinal findings are lacking. We describe a patient that developed bilateral multifocal central serous-like chorioretinopathy due to the MEK inhibitor trametinib.

## 2. Report of a Case

A 54-year-old Caucasian female presented for an eye examination prior to initiation of dabrafenib (B-raf inhibitor; 100 mg po bid) and trametinib (MEK inhibitor; 2 mg po qd) chemotherapy for cutaneous melanoma with axillary and cervical lymph node metastases. Past medical history was positive for hypertension and past ocular history was negative. Current medications included diltiazem, meloxicam, losartan, and hydrochlorothiazide. Visual acuity (VA) was 20/20 OU with normal exam findings.

**Figure 1:**
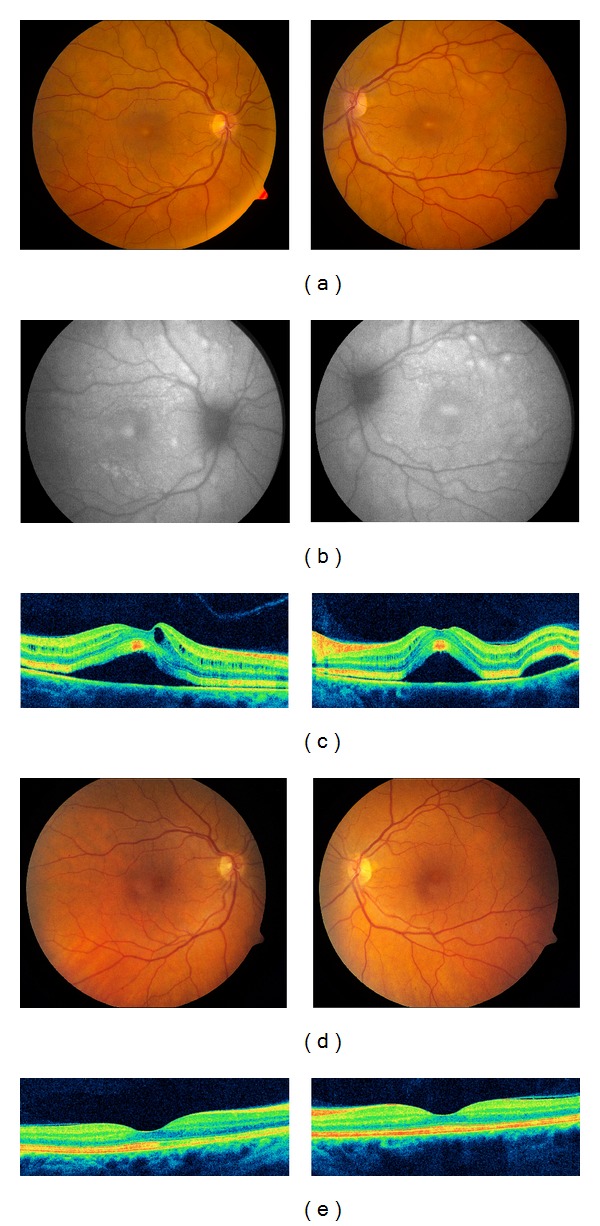
Color fundus photographs of the right and left eyes on full dose chemotherapy showed bilateral multifocal neurosensory detachments (a) seen on fundus autofluorescence as hyperautofluorescent areas (b). Optical coherence tomography (OCT) confirmed multiple neurosensory detachments with cystoid macular edema (c). After stopping chemotherapy, the neurosensory detachments resolved on fundus photographs (d) and OCT (e).

Three weeks after beginning therapy, she presented with decreased vision of 20/60 OD and 20/50 OS. Fundus examination showed bilateral multifocal neurosensory retinal detachments that were hyperautofluorescent on fundus autofluorescence (Figures 1(a) and 1(b)). Subretinal fluid (SRF) and mild cystoid changes were present on optical coherence tomography (OCT; Figure 1(c)). Both drugs were stopped and nine days later, VA improved to 20/25 OU with rapid resolution of SRF and cystoid changes (Figures 1(d) and 1(e)). Dabrafenib was resumed shortly thereafter and trametinib was restarted at a reduced dose (1.5 mg po qd) one month later. 

**Figure 2:**
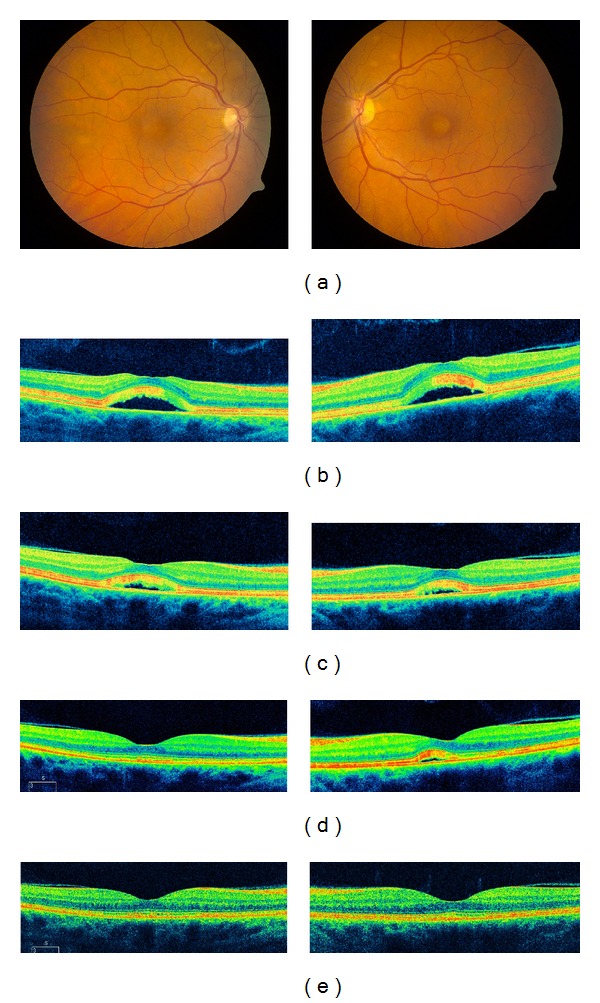
Color fundus photographs (a) and OCT (b) of the right and left eyes on full dose dabrafenib and lower dose trametinib showing recurrence of the multifocal neurosensory detachments. After stopping trametinib, OCT findings improved at 1 week (c), 3 months (d), and 6 months (e).

Four months later, her vision gradually deteriorated to 20/30 OU with recurrence of SRF (Figures 2(a) and 2(b)). Dabrafenib was continued but trametinib was again stopped with improvement of SRF one week later (Figure 2(c)). Improvement continued at 3 months (Figure 2(d)) with complete resolution by 6 months (Figure 2(e)). 

## 3. Comment

Dysregulation of extracellular signaling is an increasingly recognized factor in the development of human cancers. Three kinase enzymes are part of this pathway: mitogen-activated protein kinase (MAPK), MEK, and extracellular signal-regulated kinase (ERK) [4]. Inhibitors of each of these kinases are being investigated for malignancies. B-raf is an oncogene that is involved in the MAPK pathway. B-raf inhibition may delay or overcome resistance to MEK inhibition that may occur with prolonged therapy.

Several cases of MEK inhibitor-induced retinopathy have been reported. In a prospective, randomized, phase I/II study of a B-raf/MEK inhibitor, 2% of patients in the higher dose group developed chorioretinopathy [1]. Velez-Montoya et al. described three patients in different MEK/ERK inhibitor clinical trials who developed central serous retinopathy (CSR), one of which was multifocal [2]. In another trial, 6 patients (21%) developed central serous-like retinopathy after MEK inhibition [3]. Of those six patients, five had resolution of symptoms after dose reduction and one discontinued therapy and was asymptomatic eleven days later. To our knowledge, no cases of MEK inhibitor-induced retinopathy are published in the ophthalmic literature and consequently, the extent and severity of the retinopathy has not been previously described. While our case bears resemblance to acute exudative polymorphous vitelliform maculopathy and paraneoplastic vitelliform retinopathy, its time course (onset after starting treatment) and rapid resolution after cessation of treatment on 2 separate occasions are highly suggestive of drug toxicity.

Mechanisms underlying retinopathy in the setting of MEK inhibition are unknown, but we hypothesize that MEK inhibition results in acute retinal pigment epithelial (RPE) toxicity and dysfunction with breakdown of the blood-retinal barrier. In support of this, the MEK pathway appears to be important in maintaining the blood-retinal barrier and protecting RPE cells against oxidative and light-induced damage. Animal models demonstrate that MEK inhibition induces oxidative stress and inflammation with subsequent endothelial and blood-retinal barrier damage and RPE hyperpermeability [5, 6], providing a plausible mechanism for accumulation of SRF.

The combination of B-raf and MEK inhibition in patients with cutaneous melanoma significantly improves progression-free survival [1]. In addition to melanoma, current clinical trials are investigating MEK inhibitors for intraocular melanoma, multiple myeloma, colorectal carcinoma, thyroid carcinoma, leukemia, and other soft tissue malignancies. Consequently, the development of retinopathy may become more prevalent with more widespread use of these agents. Thus, greater awareness of this side effect may allow for prompt diagnosis and earlier cessation or reduction of dose to reduce retinopathy and vision loss in future cases.
